# Detection of susceptibility loci by genome-wide linkage analysis

**DOI:** 10.1186/1471-2156-6-S1-S18

**Published:** 2005-12-30

**Authors:** Marie-Claude Babron, Catherine Bourgain, Anne-Louise Leutenegger, Françoise Clerget-Darpoux

**Affiliations:** 1 INSERM U535, 94817 Villejuif Cedex, Villejuif, France; 2 INSERM U679, Paris, France

## Abstract

The objective of this study is to evaluate the efficacy of a model-free linkage statistics for finding evidence of linkage using two different maps and to illustrate how the comparison of results from several populations might provide insight into the underlying genetic etiology of the disease of interest. The results obtained in terms of detection of the risk loci and threshold for declaring linkage and power are very similar for a dense SNP map and a sparser microsatellite map. The populations differed in terms of family ascertainment and diagnosis criteria, leading to different power to detect the individual underlying disease loci. Our results for the individual replicates are consistent with the disease model used in the simulation.

## Background

The Genetic Analysis Workshop 14 (GAW14) simulated problem provided family data ascertained in four different populations. All members of the family were typed both with a relatively sparse map of microsatellites, and a denser map of single-nucleotide polymorphisms (SNPs). The objective of this study is to evaluate the efficacy of a model-free linkage statistics for finding evidence of linkage in the different populations using the two different maps. We also show how the comparison of several diagnostic criteria can provide clues to the underlying genetic model. This study was performed blind to the genetic model used to simulate the data provided.

## Methods

### Material

The disease under study, Kofendrerd Personality Disorder (KPD), results in an unknown combination of 12 sub-phenotypes. Families with this disorder were ascertained in four populations, with a different scheme. In 3 populations (Aipotu (AI), Danacaa (DA) and Karangar (KA)), ascertainment was based on the presence of at least two affected sibs in nuclear families, while in the last, New York (NY), large pedigrees including more than 4 affecteds were recruited. The populations differed also in the distribution of the sub-phenotypes. All family members were typed for markers on their 10-chromosome genome, without genotyping error. Two marker sets were available: 416 microsatellites spaced every ~7 cM, and a denser 917-SNP map, with ~3.5 cM inter-marker distances.

### Determination of the susceptibility loci

To identify the number and location of the susceptibility loci involved in the simulated disorder, a pooled linkage analysis of all 100 replicates in a given population was performed with the nonparametric linkage (NPL) statistics [1] using the ~7-cM microsatellite map and the ~3.5-cM SNP map. The affection status for KPD was as indicated by the clinicians in each population. The NPL was calculated using ALLEGRO [2] for the 3 populations AI, DA, and KA. Due to large family sizes and memory limitations, the NPL for the NY population was obtained using GENEHUNTER [1] using the microsatellite map only. The NPL statistic was chosen, as the overall value can easily be calculated from the results of the individual replicates.

### Determination of the threshold under H_0_

After the pooled analysis, some chromosomes appeared not to harbor any susceptibility loci. Five chromosomes (see "Results") represented the null hypothesis of no linkage, whatever the population, giving a total of 1,500 replicates simulated under H_0 _(5 chromosomes × 3 populations × 100 replicates). Because of its ascertainment scheme, and computing limitations, the NY population was ignored in this step.

The value of the maxNPL that was exceeded in 0.5% of these 1,500 replicates, was then determined. It corresponds to the threshold for declaring linkage at the 5% genome-wide level, after a Bonferroni correction for 10 independent chromosomes.

### Power to detect linkage in individual replicates

This was calculated as the number of replicates in which the value of the NPL at the putative disease locus exceeded the 5% genome-wide threshold value.

## Results

### Determination of the susceptibility loci

Four, and possibly 5, linkage regions had evidence of linkage by the pooled analysis, as shown in Table 1, where the marker giving the highest NPL score in each region is reported for the microsatellite and SNP map.

Apart from the region on chromosome10, whatever the diagnosis criteria, one can conclude there is a susceptibility factor on chromosome 1, 3, 5, and 9. For these chromosomes, in all populations, the peak occurred at the same marker or the one immediately adjacent.

In addition, the different results obtained in the populations AI, DA, and KA, which only differ by the definition of the affection status, show that the genotype-phenotype relationships vary widely across populations. This is well illustrated for chromosome 9, where the NPL ranges from 8.6 in DA to 37.9 in KA.

In population AI and NY, the diagnosis criteria seem to be the same, as indicated evidenced by the similar distribution of sub-phenotypes among cases, but the ascertainment criteria and family structures differ. The NPL values are greater for the AI nuclear families than for the NY extended pedigrees for chromosome 1, 5, and 9, whereas they are similar for chromosome 3. This result is interesting in view of the debate "sampling large extended pedigrees vs. smaller familial structures". Here, we show that for the simulated model, two nuclear families with two affected sibs are more informative than one three-generation pedigree with four affected members.

For chromosome 10, the signal is very weak because this NPL value was obtained for 10,000 families in AI, DA, and KA and 5,000 families for NY. So this could well represent a factor with an effect difficult to detect by linkage analysis or a factor observed only in a subgroup of affecteds.

Similar observations were obtained in a pooled analysis using the denser SNP map. It was not possible to align the two maps, because no indication was given about merging the two maps. However, the peaks were located about the same distance from the first marker of each map.

### Determination of the threshold under H_0_

For the 5 chromosomes (2, 4, 6, 7, and 8), the highest NPL obtained on the pooled data for the microsatellite map was 2.65. As explained in the "Methods" section, these 5 chromosomes were considered to carry no risk factor and were thus presumably simulated under the null hypothesis of no linkage. It is thus possible to establish the 5% genome-wide threshold from the distribution of the NPL scores observed in the individual replicates of the three populations AI, DA, and KA. This threshold was found to be NPL = 3.3 and 3.2, using the microsatellite and SNP maps, respectively.

### Power to detect linkage in individual replicates

The power to detect linkage in the 5 regions found by the pooled analysis is given by the number of replicates for which the statistic value is over the 0.5% threshold, as shown in Table 2 for the microsatellite and SNP maps. Both maps provide very similar power. Indeed, power depends on the amount of information on the resemblance between affected individuals that can be extracted from the marker data. Both maps have very similar information content, with an average of 0.91 and 0.81 for the microsatellite and SNP maps, respectively. The smaller heterozygosity of the SNPs vs. microsatellites (0.34 vs. 0.76) is compensated by the higher density of the map. The power observed in Table 2 reflects the magnitude the NPL observed in Table 1: regions having high NPLs in the pooled analysis are more easily detected in the individual replicates.

## Discussion

### Before knowing the simulation model

The detection of the different risk factors varies according to the diagnosis strategy and the chromosome, giving clues on the genetic basis of KPD. Let A denote the anxiety-related symptoms, B, the behavioral, and C the "communally shared emotions" sub-phenotypes. From the indication given to all participants prior to the analysis, individuals in AI are declared affected if they have A or B or C symptoms, while in DA, B is prominent. In KA, only those individuals with either A or C, whatever their B symptoms, are classified as affected, while those with prominent B symptoms are not.

The chromosome 1 risk factor is very well detected in DA, and not in the other populations, suggesting that it is involved only in the determination of behavior B. On the other hand, the risk factors on chromosomes 5 and 9 do not seem to play a role in determining B (lack of evidence in DA), but are probably involved in the determination of A and C.

Chromosome 3 is detectable in all populations, with varying intensities. It is probably involved whatever the diagnosis criteria. However, in the AI population, this locus is detected in 41% of the replicates, but the NPL values range from 1.38 to 5.71. This observation is true even when the power is high, such as in the DA population where the values range between 1.66 and 6.13. This risk locus illustrates the difficulty of replicating an earlier linkage finding, as shown by Clerget-Darpoux et al. [3].

Finally, the chromosome 10 risk factor is never detected with sample sizes of 100 families. As we have seen in the pooled analysis, it is a factor difficult to detect by linkage analysis. Note that it was detected in DA and KA by association analysis [4].

### After knowing the simulated model

The disease model used in the simulation was given during GAW14. Four disease loci and two modifier genes were simulated, and their position on the SNP map was given. Neither D5 nor D6, which act as modifier genes involved in the phenotype P2 that regroups most of the traits defined as behavioral related traits B, are expected to be detectable by linkage analysis, even with the large sample size of the pooled analysis. In fact, disease locus D6 was not detected at all. The other loci were all detected at their exact location on the SNP map, with the exception of D2 on chromosome 3 in DA (maxNPL found at the adjacent SNP) and D5 on chromosome 10 in AI (maxNPL located 16 cM more centromeric). The value of these two maxNPL are given italics in Table 1.

Analysis of the individual replicates gave results consistent with the disease model used in the simulation. In population DA, individuals are declared affected when they have phenotype P1, determined by the two loci D1 and D2 on chromosome 1 and 3, respectively, with a highly penetrant dominant mode of inheritance. These two loci are therefore easily detected in this population.

Locus D2, on chromosome 3, underlies all the phenotypes. This explains why it is very well detected in all 3 populations, whatever the ascertainment criteria. In contrast, locus D3 on chromosome 5, and locus D4 on chromosome 9 determine phenotype P2 and/or P3. This explains the high level of detection in KA and AI, but not in DA. Note that D4, which acts in a recessive manner with a high penetrance, displays more evidence of linkage than D3.

The answers also provided some explanation of the difference in magnitude of the maxNPL in the pooled analysis of the AI and NY replicates. The ascertainment criteria were not only different; but showed greater heterogeneity in NY. In the NY study, the 4 affected individuals could each have different phenotypes, determined by different combination of the disease loci, thus lowering the resemblance between affecteds and the expected value of the linkage statistic.

## Conclusion

In this simulated problem, the results obtained in terms of detection of the risk loci, threshold and power were very similar for the microsatellite and SNP map. A sparser map, with very polymorphic markers, brings as much information on the IBD sharing than a denser, less polymorphic marker map, at a smaller genotyping cost. Whether this is true in all cases remains to be explored. However, this point should be kept in mind before embarking on a genome scan using SNPs.

The power to detect linkage varies according to the population diagnosis criteria and to the disease locus.

## Abbreviations

GAW14: Genetic Analysis Workshop 14

IBD: Identical by descent

KPD: Kofendrerd Personality Disorder

NPL: Nonparametric linkage

SNP: Single-nucleotide polymorphism

## Authors' contributions

M-CB performed the analyses and drafted the manuscript. CB and A-LL provided the file formatting programs. FC-D and M-CB designed the study. All authors read and approved the final manuscript.

## Figures and Tables

**Table 1:** Regions detected in the analysis of the pooled 100 replicates.

Chr	MS	AI	DA	KA	NY	SNP	AI	DA	KA
1	D01S0023	21.60	41.65	12.18	14.59	C01R0052	21.53	47.08	13.43
3	D03S0127	30.32	37.70	26.91	30.16	C03R280		35.19	
						C03R281	28.93	*35.11*	28.86
5	D05S0172		8.52		22.11	C05R0380	25.57	7.65	34.40
	D05S0173	26.98		35.24					
9	D09S0347	24.61	8.61	37.88	11.17	C09R0765	23.51	8.11	34.01
10	D10S396	2.29				C10R0874	1.61		
	D10S400		6.59	5.67		C10R0880	*1.06*	8.07	6.49
	D10S408				0.73				

Highest NPL and corresponding marker of the microsatellite (MS) and SNP maps. The value of the maxNPL at the true location for DA on chromosome 3 and AI on chromosome 10 are given in italics.

**Table 2:** Number of replicates for which the statistic value is over the 0.5% threshold for the 5 regions, using the microsatellite (MS) and SNP maps.

		MS map			SNP map		
		
Chromosome	Locus	AI	DA	KA	AI	DA	KA
1	D1	7	84	5	16	96	6
3	D2	41	69	29	36	68	30
5	D3	26	1	54	22	0	60
9	D4	21	2	70	18	0	59
10	D5	0	1	0	0	0	0
